# Cholera, British seamen and maritime anxieties in Calcutta, c.1830s–1890s ‘*The William Bynum Prize Essay*’

**DOI:** 10.1017/mdh.2021.25

**Published:** 2021-10

**Authors:** Manikarnika Dutta

**Affiliations:** Oxford Centre for the History of Science, Medicine and Technology, University of Oxford, Oxford, UK

**Keywords:** Cholera, British seamen, Calcutta, Sanitation, Maritime hygiene, Colonialism

## Abstract

From the mid-nineteenth century, seamen were increasingly identified as vectors of epidemic diseases such as cholera. The rising acceptance of the germ theories of disease and contagion and the transition from sail to steam at this time increased the fear of the rapid spread of contagious diseases through these mobile people. This article examines how the British naval authorities, ship surgeons and the medical and municipal authorities in the Calcutta sailortown sought to improve maritime health and hygiene to prevent the spread of cholera among and by British seamen. Nineteenth century Calcutta is an ideal context for this study on account of its epidemiological notoriety as a disease entrepot and the sea route between Calcutta and British ports was one of the most closely monitored for disease in the Empire. The article argues that a study of cholera among British seamen can generate important insights into the relationship among disease, medicine and colonialism and in doing so shed light into a neglected aspect of the history of nineteenth century cholera, the British anxiety regarding disease dispersion, practice of hygiene and sanitation and British seamen’s health.

## Introduction

There is a City in the east, its atmosphere is laden with malaria, its river is the reservoir of a deadly poison. Within its dwellings abide the seeds of a fruitful mortality, the harbour of the city is the Maelstrom of death. We need hardly add that the City is Calcutta… Years after year the brave sons of the sea are engulfed and perish in its vortex… Our metaphor points to that stern reality, the poison of cholera, which, discharged at various points in the shape of sewage upon the river-bank and into the centre of the stream, pollutes the water and the air; and, added to other malignant influences, common to all rivers in malarious countries at remote distances from the sea, converts a haven of refuge into a port of danger.^1^

This macabre report from the *Indian Medical Gazette* highlighted cholera as one of the main causes of mortality among British seamen in mid-nineteenth century Calcutta. Seamen’s health in the tropics had been a contentious issue for the British Empire since the mid-eighteenth century. As seamen had an important role in trade and war, their poor health could jeopardise Britain’s imperial ambitions. The British naval authorities were concerned with their health, hygiene, diet, discipline and the adverse impact of the diseases of the ‘warm climate’, but they were widely criticised across the empire for not doing enough for seamen’s welfare.^2^ Merchant seamen in particular were victims of apathetic shipowners and lack of strict sanitary regulation. Attention to their health increased in the 1830s due to their alleged role in disease dispersion. A ship from the Baltic regions was suspected to have brought the disease to Sunderland in 1831, starting the first pandemic in Britain. Dr William Macmichael suggested that cholera prevailed in waterside communities and usually began after the anchoring of ships with diseased crew, indicating the contagious nature of the disease.^3^ Seamen’s image as potential carriers of disease was further solidified with the popularity of the germ theories of disease in the 1850s and the first spread of a cholera epidemic from Asia to Europe through sea routes in the 1860s. Seamen were now increasingly held responsible for spreading the cholera pathogen and their living conditions and mobility came under close surveillance.^4^ On board ship, infection spread more rapidly, depleting manpower.^5^ Ship surgeons and captains managed seamen’s health and supervised diet and sanitation. On land, officials were able to control port outbreaks by quarantining seamen and their infected companions.^6^ Treating port areas as ‘gateways of disease’ and ‘portals of death’, port and municipal authorities implemented several sanitary reforms.^7^

In studying cholera among British seamen in Calcutta and in transit to and from the city, this article generates important insights for our understandings of the dynamics among disease, medicine and colonialism in the nineteenth century. Calcutta’s importance as a case study is derived from its popular representation as a veritable breeding ground of cholera.^8^ The city, known to be a major graveyard for British seamen where as many as 10% of the seamen in transit was estimated to die, was a major focus of sanitary reforms.^9^ Indeed, for most of the nineteenth century, medical practitioners and officials in Britain and India treated cholera as a ‘disease of locality’, and identified Bengal as its ‘home’ and Calcutta as the epicentre of choleraic disease and death in the world.^10^ In 1850, a report comparing Bombay and Calcutta said, ‘Bombay lays lower, or as low, is more densely populated in a smaller space, and worse drained than Calcutta, and has stagnant marshes to the north-east of it, and yet medical men tell you that there is no cholera amongst European crews there’.^11^ Consequently, government officials in British India were most cautious regarding seamen transiting through Calcutta. One of the busiest ports in the British Empire in the mid-nineteenth century, the city also had the largest sailortown in Eastern Asia, which makes it an ideal context for this study. Between the 1830s and the 1890s, maritime sanitary regulations placed great emphasis on cholera, starting with the first cholera epidemic in Britain and diminishing when wide acceptance of the cholera bacillus led to the standardising of preventive measures on an international scale. A study of this period allows the article to track the shifts in maritime medicine in the tropics and the British Empire’s changing perception of seamen’s importance.

Cholera has been central to the historical understanding of the relationship between disease, medicine and colonialism in the British Empire and beyond. Despite its intermittent nature, the disease received more attention from British medical practitioners and imperial government in the nineteenth century than perennial problems such as fever and tuberculosis.^12^ Historians have extensively studied cholera epidemics on land in order to understand the impact of disease, quarantine, travel and immigration upon the making of imperialism and colonialism and how cholera recast the understanding of certain objects, customs, climates and landscapes.^13^ Relatively few historians have examined the history of surgeons and passengers on board cholera-affected ships and in port towns, and even fewer have explored the state of seamen as vectors and victims of cholera.^14^ In spite of the large amount of evidence intimately connecting seamen with travel networks, disease dispersion and empire-making in the nineteenth century, historians have at best mentioned them in passing in their explorations into the relationship between disease, medicine and imperialism.^15^ This article corrects these oversights and places maritime medicine and management of cholera at sea and in ports at the centre of our understanding of the relationships among disease, medicine and colonialism.

In this article, ‘British seamen’ refer to the white crew in the British naval and merchant services, comprising seamen from various European nations such as England, Scotland, Norway, Sweden and so on. The rationale and criteria for British nationality for migrant workers changed several times through the eighteenth and nineteenth century.^16^ Many seamen were described as European seamen rather than as British citizens in naval documents. Government records, medical journals and books on cholera rarely mentioned the health of lascars and other black and Asiatic seamen.^17^ The article begins with a study of the opinion of medical practitioners on seamen as propagators of disease. The next two sections study methods of preventing cholera on board ships. Cholera prevention in the nineteenth century has been extensively explored from the perspectives of land-based practitioners and state-governed sites such as hospitals, prisons and barracks, but the relationship between cholera and maritime medicine and hygiene has not been understood until now. By summarising the major therapeutic models, this article identifies the importance of ships as mobile laboratories for cholera treatment and medical innovation. In doing so, it analyses how treatment on ships differed from treatment on land and the extent to which these measures were a key component of maritime medicine. The final two sections examine the British colonial state’s sanitary reforms to protect British seamen on board ships anchored in Calcutta and in their living quarters near the port of Calcutta. As official anxieties regarding cholera epidemics escalated in the second half of the century, especially after the cholera pandemic of 1866, the colonial state took greater interest in sanitation and regulating the quality of the water and alcoholic beverages consumed by British seamen.

A study of the prevention and reform measures, the article argues, enhances our understanding of the entangled history of public health, preventive medicine, seamen as agents of imperialism and colonial power in the context of nineteenth century British India. A look at both land and sea enhances our understanding of the regulation of British seamen, colonial anxieties regarding maritime spaces and the impact of cholera treatment to British maritime hygiene. It is important to note that all nineteenth century accounts of cholera among seamen emphasise the specific vulnerabilities of ‘white’ seamen to the disease allegedly originating in India. This was consistent with the racialising of India’s ‘toxic’ environment as harmful to European bodies in a way precluding long-term habitation and endangering their racial quality. This article does not have the scope to delve into how issues of race informed cholera treatment and sanitary measures, and how the changing nature of the concept of race itself transformed medical practices. However, it acknowledges the existing research that shows how sanitary regulation of Calcutta was motivated by a certain form of racial control of particularly white bodies through dietary regulation and segregation from the society’s non-white underclass.

## British seamen as propagators of disease

This section provides an overview of the context for this study of cholera among British seamen, the history of medicine and British colonialism in India. British seamen’s complicity in the spread of cholera started crystallising in the 1830s when contagionism was gaining in popularity as a medical opinion. Dr James Kennedy of the Royal College of Surgeons in London illustrated this opinion through the example of the *Carnatic.* After the ship sailed from Madras in 1830 with several crew members who had recovered from cholera, the disease broke out during the voyage and afflicted many seamen including the recent victims. Kennedy surmised that the cholera pathogen, till then thought to be the result of miasma generated by animal or vegetable effluvia, must have been transmitted through the human body to travel deep into the ocean from the shore.^18^ He also mentioned that shortly after its beginning in Calcutta in 1817, cholera had spread in a number of the East India Company’s vessels at Sagar Island. Several seamen died within a week after the first death occurred on board *Warren Hastings* on 7 September 1818, while other ships had fewer cases.^19^

In recognition of cholera’s transmissibility from one body to another, the British government implemented some sanitary measures on board ships based on the prophylactic virtues of a disciplined life – diet, hygiene and comfortable living condition. In the first half of the nineteenth century, land routes were monitored closely as the pandemic in the 1830s seemed to have spread to Europe through Russia rather than the Atlantic or Mediterranean. Despite the frequency of cholera among seamen, the British naval authorities thought it unlikely for the cholera miasma or pathogen to spread through the slow and often roundabout journeys made by sail ships. They quarantined ships carrying cholera-infected crew and passengers outside the harbour for up to 60 days. Quarantine spoiled organic cargo, led to shortage of food on board incoming ships and was financially ruinous for shipowners. Merchant communities risked the ill-repute of undermining public health and opposed the system on the ground of its adverse impact on trade. Many cities replaced quarantine with sanitary measures to keep urban spaces clean and prevent contagion.^20^ In the 1840s, the popular opinion turned against quarantine with increasing preference for sanitary reform of public spaces and a humanitarian interest in the welfare of seamen.^21^ The growing support for miasma rather than the germ theory of disease dispersion, the political resistance to quarantine and a lack of understanding of cholera, promoted the theory of anticontagion.^22^ ‘Is it a fungus, an insect, a miasma, an electrical disturbance, a morbid off-scouring of the intestinal canal?’, asked *The Lancet* in 1853.^23^ On account of its misunderstood aetiology and frequent misdiagnosis, using cholera data from the nineteenth century presents a challenge of misrepresenting the extent of the disease. This article recognises this problem and discusses the discourse rather than statistics of cholera.

The British shipping industry’s acknowledgement of seamen’s vulnerability and role in spreading cholera was not translated into international awareness of the problem. The discussion on cholera at the eight International Sanitary Conferences between 1851 and 1894, which marked the first attempt to address and contain the ‘propagation of disease’ through international co-operation, was centred on passengers rather than seamen. In the 1850s, long-distance commercial steamships in the Indian Ocean halved journey times in most routes and hastened the spread of disease.^24^ Larger ships accommodated more people than before, thus compounding the possibility of spreading germs through contagion.^25^ In 1865, cholera approached Europe for the first time by sea instead of land routes in the aftermath of an epidemic during the Hajj pilgrimage in Mecca. The allegation that Indian pilgrims transmitted the disease to Arabia led the British Empire to start regulating Muslim pilgrimage for the first time.^26^ The pandemic reinvigorated discussions on the risk to seamen’s health and seamen propagating cholera through contagion. In the following year, the third International Sanitary Conference held in Constantinople discussed how infected seamen and passengers carried cholera to Egyptian and Mediterranean ports and subsequently to Europe.^27^ Seamen’s and immigrant labourers’ increasing mobility intensified the threat of global disease dispersion, thus making their health a global concern.^28^ Steamships enabled cholera victims to travel long distances before beginning to show symptoms. Various international hygiene agreements were signed in this period to regulate the movement of merchant seamen.^29^

By the third quarter of the nineteenth century, seamen were widely recognised as potential carriers of epidemic cholera, plague and yellow fever that led to innumerable deaths across the world. Dr Heber Smith, surgeon at the United States Marine Hospital in New York, wrote that if the ocean was the great highway of commerce and disease, seamen were highwaymen who robbed individuals and communities of their health. He also remarked that the simplest sanitary measures could prevent these diseases, but the shipping industry rarely gave due importance to sanitary regulation for ships and seamen. He put great faith in the ‘influence and catholic scope of sanitary science’ to ameliorate seamen’s physical condition and protect the health and lives of both seamen and land people.^30^ Governments needed to prioritise sanitary regulation to prevent seamen from disseminating ‘cholera, small-pox, typhus, yellow and relapsing fevers, and particularly of venereal diseases’.^31^ Except for few studies such as this, seamen remained marginalised in medical texts and government records and usually not compared or even mentioned in discussions of the health of itinerant peoples.

In 1876, influenced by sanitary conferences, medical studies and commercial needs, the British government implemented maritime quarantine and a number of sanitary measures in several colonial Indian port cities and passed the Native Passenger Ships Act for British ships carrying pilgrims.^32^ The anxiety of preserving colonial trade, overseas possessions and imperial prestige and the fear of the tropical environment and poisonous food and drink played a significant role in the increased monitoring of seamen’s health. The public complaints about the neglect of seamen continued even after the passing of several Merchant Shipping Acts and their amendments from the mid-nineteenth century onward. Regardless of naval reforms, merchant shipowners infringed the rules of providing adequate lime juice, fresh vegetables and good quality food, clean water, well-ventilated and hygienic quarters and regular wages to seamen throughout the nineteenth century.^33^ Ship surgeons and captains shouldered the responsibility of treating ill seamen with the equipment and medicines recommended by the naval authorities. They were compelled to improvise medical provisions in the absence of adequate resources to care for cholera patients in their workspace below the deck or the forecastle, leading to innovations in British maritime medicine that the next section will exaplore.

## Ship surgeons and cholera treatment

In his article on the treatment of cholera in the nineteenth century, historian Norman Howard-Jones called cholera therapy ‘tragicomic’ and the most ‘grotesque chapter’ in the history of therapeutics.^34^ He thought cholera therapies were mostly ineffective and harmful to patients. Cholera was manifested through diarrhoea and vomiting and as such led to loss of water and electrolytes from the body. Bleeding, initially the most favoured treatment of cholera, especially in British India, compounded the problem. Other methods included the use of calomel and opium, cauterising patients with blisters along their spine, baths of hot or cold water, pumping air into bellies through anus, electric shock and intravenous infusion of saline water. Howard-Jones points out that saline water alone had some potential of success by replenishing the lost fluid. Most of these methods were derived from the humoralist idea that patients’ bodies needed to be cleansed of the cholera-causing substance.^35^ Numerous articles in British medical journals and treatises were devoted to studies of cholera treatments. In the hospitals in Britain and British India, treatment of cholera depended upon the surgeon’s understanding of the origin of cholera. The British Navy used a standard protocol practiced by qualified surgeons on board. The British naval administration issued medical guides to captains of merchant vessels without surgeons on board. These guides provide an account of the transformation of treatment of cholera among merchant seamen from mid- to late nineteenth century.

*The Seamen’s New Medical Guide* (1842) gave a comprehensive step-by-step method of bringing diarrhoea and cholera under control on board ships. The book urged taking diarrhoea seriously as it was usually the first symptom of cholera.^36^ It recommended strong doses of diarrhoea medicine for cholera patients: brandy during the attack and half a tumbler of mulled wine with a small quantity of toasted bread and castor oil after the symptoms subsided.^37^ The insistence on basic food and drink was soon considered inadequate, for a guide book from 1845 recommended bleeding, blankets, hartshorn liniment, calomel, a draught containing ten or fifteen drops of laudanum and two tablespoon of brandy in a small quantity of water and occasional doses of castor oil and light nourishing diet.^38^ Not only were medical items added, the book also made some observations on the general state of cholera patients. It noted that patients usually lived if they survived the first 4 or 5 days. On their recovery, it instructed, cheap and dispensable articles including rags, cordage, paper and old clothes were to be burnt, and more expensive items such as clothing, furniture and wooden articles cleansed by a strong mixture of soap and water.^39^ These recommendations were derived from the methods doctors preferred during the 1830s cholera pandemic in Britain.

Before British medical practitioners identified and suggested ways of destroying the source of pathogenic contamination, they advocated various contradictory methods of treatment. Dr John Snow’s method of therapy in the 1850s reflected his transition from humoral to germ theory of disease. He prescribed destroying the cholera particle by chemical substances like olive oil, animal charcoal, sulphur, oil of cajeput, camphor and creosote or saline intravenous injection when the patient was in a state of collapse.^40^ Those who subscribed to the traditional humoral theory, such as the surgeons in St Thomas’ Hospital in London, used the mixture of half pint water, two grains tartar emetic and half ounce sulphate of magnesia as medicine and lead, opium, turpentine, carbon, lime juice, burnt coffee, soda water, hot air baths and ipecacuanha for treatment.^41^ These were not very different from the therapeutics used in the British Navy till the 1860s. Naval surgeons based their method of treatment on both humoral and nervous theory till at least the mid-nineteenth century.

The emphasis on chemical substances to supplement healthy food and cleanliness increased in the later decades. In his 1854 book, Dr Gustavus Horner suggested using magnesia and supercarbonates of soda as a remedy for nausea and acidity; taking one to three grains of the powders of calomel and opium, the sweet spirits of nitre, sulphuric ether, laudanum and essence of peppermint as anodynes and antispasmodics; having hot baths of seawater with pulverised capsicum, putting mustard and blisters on one’s body and sprinkling powdered sulphate of Quinia on the skin; drinking hot brandy toddy and a julep of carbonate of ammonia, Gum Arabic, sugar and water a stimulant and applying hot water bottles on hands and feet.^42^ He advised a diet of drink of rice and barley water for cholera patients. During convalescence, patients were to be given powdered cantharides boiled in spirits of turpentine, which was to be replaced with acetate of lead if it acted a strong sedative. The book suggested quinine, an infusion of Columbo root, and a concoction of the root, powdered cinchona and ginger for restoring patients’ strength.^43^ The extent of medication shows merchant seamen’s health was now taken more seriously and medicine chests were better equipped. *The Seaman’s Medical Friend*, published in 1857, emphasised isolating the patient, throwing his excreta overboard, fumigating or whitewashing his sleeping place after recovery and boiling his bedding and clothes before they were used again.^44^ Finally, the Navy-authorised guide by Harry Leach recommended using diarrhoea mixture (a combination of Diarrhoea Powder, laudanum and water) after every bowel movement, no medicine during cramps, and nothing but nourishing diet during fever.^45^ The reduction in the number of recommended medicines also points out the growing certainty over the method of treatment after years of experimentation. The treatment recommended in books published in the 1850s and later differed significantly from land-based hospitals.

Ship surgeons innovated remedies depending on the availability of resource as they had access to few of the apparatus available to land-based hospitals. They relied on accessible materials (calomel, opium, hot and cold water) and sanitation to nurse cholera-affected seamen.^46^ As severe cholera caused death in fewer than 10 hours, they did not have enough time to observe patients and experiment on the dose and composition of decoctions. In fact, they were at great risk of succumbing to cholera on account of their proximity with cholera-stricken seamen and passengers. For example, the assistant surgeon on board *Fox* died of cholera in 1852.^47^ Sir Charles Forbes, chartered for service against pirates in Burma in the 1850s, wrote about his experience of getting cholera,

Visit the fort, call on friends, see Sillar, find no money sent from Hong Kong, am in distress as to what I take up to China. Tiff at Bank and proceed to ship; am feeling very seedy; coldness about the stomach and general lassitude; go to bed, at midnight wake up with fearful gripping pains; bear it till 2 a.m., 31st, when I vomit and am purged; send for surgeon of ‘Pottinger’; says I have Cholera, am drawn together with cramps, quite cold and vomiting frequently… the day is spent in thinking of the uncertainty of life and how soon we are cut off and gone. (Bombay, 30–31 August 1854)^48^

In the late nineteenth century, ship surgeons were hopeful of curing cholera before the patient was in a state of collapse. Some of them used hypodermic injection of chloral or quinine to seize the morbid process. They resorted to ad hoc measures such as using a chest as a surgery table and substituting a known emetic with another in case of short supply. Surgeons on shore used warm blanket to sustain the patient’s body temperature and gave them iced drinks. After the mid-nineteenth century, they stopped giving stimulants since their injurious impact on kidney and liver was well known by then and the nervous theory of disease behind such treatment was no longer dominant. Surgeons used stimulants sparingly for circumstances such as restoring the patient’s heartbeat. They used astringents such as chlorodyne and diluted sulphuric acid, sinapism and diluted hydrocyanic acid to cure gastric irritability caused by diarrhoea. Careful nursing such as applying heat on the patient’s body and rubbing his limbs was essential.^49^ The distinction between the two methods of treatment from different quarters of the nineteenth century exhibits how ship surgeons modified their approach to treatment in keeping with the transformation of theories of disease. They relied more on sanitation to prevent cholera on board ship. The tropics became a contentious point and a convenient excuse for the limitation of medical and sanitary methods in the debate about the origin of cholera. However, incidents of cholera outbreak gave ship surgeons the opportunity to test recent developments in the fields of the science of hygiene. Their observations on atmospheric impacts on the body, the problems of congestion and the possibility of contagion prepared a field for complementing physiological studies in Britain and elsewhere, though the question of how is beyond the scope of this article.

## Sanitary reform and cholera prevention on ships

The mortality statistics reported in annual accounts of British naval health do not reveal the impact of medicine on cholera, as cholera was often classified as diarrhoea or another disease owing to the lack of understanding of its aetiology. This section will examine the research presented by the Inspector-General of Hospitals Robert Lawson regarding the outbreak of cholera in ships at sea at a meeting on 10 June 1871. Lawson’s article offers a comprehensive overview of the circumstances of cholera and sanitary measures in ships that travelled from Gravesend to India in 1866. Lawson outlined three causes of epidemic diseases: general, locality-specific and individual-centred. On *Windsor Castle*, a large ship with a cargo of railway iron, an artilleryman got diarrhoea or cholera after the start of the journey, to be followed by other passengers and seamen. Nine persons died before the ship passed the Cape of Good Hope, after which health on board the vessel improved, and no further death was reported. Dr Hanrahan, the ship’s staff-assistant and surgeon, had the latrines thoroughly cleansed with distilled water and sprinkled with chloride of lime every 2 hours, the decks fumigated with nitric acid fumes, the lower deck scraped, scrubbed and sprinkled with chloride of zinc and lime, the ventilation shafts cleaned and the passengers kept on deck for as long as possible. He had all the belongings of the deceased thrown overboard and their berths scrubbed and washed with chloride of lime. The logs of *Lord Warden* showed a similar pattern of cholera dying out once the ship passed the Cape.^50^ Cholera was presumed to be at its severest in the tropical region and its intensity depended on fluctuation of temperature. Other reports, however, point out that cholera did not always occur when the ship moved from colder to warmer climate. In one instance from 1875, cholera broke out on board *Glasgow* as it moved towards Calcutta from Trincomalee and the temperature dropped almost 20°F.^51^ The relationship between cholera and warm climate, therefore, remained a matter of debate till the last quarter of the nineteenth century, and ships were the best contexts for outlining this epistemological problem due to their mobility.

Other considerations for the occurrence of cholera included poor diet and spread of infection during a halt at a port. The ship *Newcastle*, journeying from India to England in 1866, was seized with cholera between the Cape and St Helena in the South Atlantic Ocean. In 1867, nearly 60 seamen and officers suffered from malignant cholera and choleraic diarrhoea during HMS *Jumna*’s passage to India. Lawson observed that those who ate the beef and bread procured in England were more vulnerable to the disease. Drawing examples from other books, Lawson mentioned several cases from the 1850s to illustrate his point. A ship named *Sultany*, which sailed from Calcutta on 10 February 1854 with eighty crew members and 375 passengers, had thirty deaths by the time it reached Mauritius on 24 March. In 1859, the P&O Company’s steamship *Oriental*, carrying 588 passengers, mainly troops, left for Ceylon from Bombay when cholera raged through the city. Twelve deaths were reported by the time the ship anchored at Galle as an emergency stop. The disease had subsided by the time the ship reached Mauritius.^52^

Lawson also referred to Dr Murray’s report on treating cholera on board *Gertrude* that transported 120 military invalids from Calcutta to England in 1859.^53^ He remarked that the observations made in the report differed from the official report submitted by the ship’s military medical officer to the Director General of the Army Medical Department and the ship’s log sent to the Registrar General of Seamen, in particular the description of the disease that killed nine and incapacitated many soldiers. In 1864, passengers of *Queen of the North* met with bowel ache and diarrhoea, which the medical officer ‘hesitated to call cholera’. The more severe cases were termed as cholera, which claimed twenty-four lives. The crew of the ship, who lived in the forecastle and did not go into the hold, did not get cholera, but the chief officer, who apparently drank water in large quantities, died of cholera.^54^ Finally, Lawson wrote about five cholera deaths on board *Durham*, which travelled from Calcutta to London in 1866. The ship’s medical officer reported that nearly half the seamen slept on the top deck owing to the heating of the lower deck, and none of them had cholera.^55^ He concluded that cholera spread like a wave, without specifying the mode of circulation, and supported the tropical origin theory despite having surveyed cholera cases originating in England.

Over the next 20 years, Lawson collected a massive amount of data about cholera on board ships. In a presentation to the Epidemiological Society of London in 1891, he cited the Sanitary Commissioner for the Government of India’s report for 1881, which contained a detailed account of cholera on board ships transporting coolies from Calcutta to Mauritius, Natal and the West Indies from 1871 to 1880. Out of the 222 ships carrying 129 527 coolies, thirty-two ships were affected by cholera and 181 persons died.^56^ The report did not specifically mention any sailor’s death, though it would be unlikely for not one among the crew dying. In the 1880s, the naval authorities were optimistic of eradicating cholera from naval vessels to a large extent and claimed to have learnt a great deal about the method of cholera transmission.^57^ The cholera epidemic in 1891, which affected forty-four seamen and killed twenty-three in the ships *Redbreast* and *Marathon* in Bombay between 11 and 15 September, belied this optimism. Vindicating Lawson’s belief that cholera in the port city was the precursor to cholera on board ship, the crew of these ships fell ill after going to the shore, where they supposedly contacted the disease from the local people. There was no sign of the disease in Karachi and Trincomalee, the ports these ships arrived from. Cholera on board was particularly dangerous since infected persons could not be effectively isolated. Some surgeons also believed that the depression of watching one’s colleague die made seamen even more likely to succumb to cholera.^58^ The estimated deaths from cholera in the second half of the nineteenth century does not suggest any decline in the disease’s virulence.

Lawson’s reports indicate uneven success of sanitary reforms to prevent cholera and the inconclusive nature of medical intervention on board ships to and from Calcutta. Most doctors were attentive to the disruptive influence of weather fluctuation and the ship’s changing architecture on seamen’s body. Some of them wrote about the success of sanitation but the steady number of annual cholera cases probably indicate that some ships, especially those without surgeons on board, neglected prescribed measures, and sometimes none of the measures worked against cholera. In 1870, Gavin Milroy, member of the Epidemiological Society of London, noted that despite the study of epidemic cholera achieving ‘great national importance of epidemiological inquires’, the knowledge generated in the past 50 years or so was not sufficient in preventing and curing it.^59^ The Merchant Shipping Act of 1867 was revised in 1894 as it had not adequately improved seamen’s conditions. The new Act emphasised improvement in shipbuilding, saying all crew spaces should be securely constructed, properly lighted and ventilated so that seamen are protected from the weather and sea.^60^ The results, not so encouraging, were criticised by government officials. In addition to medical and sanitary intervention on board ships, hospitals in port cities treated cholera-affected seamen and sanitary reforms were undertaken to protect seamen from cholera.

## The treatment of cholera on shore

The British colonial government implemented a number of sanitary regulations in the areas frequented by seamen. Its reformist and development strategies could be seen as part of the broader impulse of domesticating India’s environment, perceived as an epidemiological threat for European settlers. In the beginning of the nineteenth century, the public works departments in colonial cities in India had started building wide roads in densely populated areas to ease out congestion-related health problems.^61^ The Lottery Committee in Calcutta was a product of the sanitarian need to protect European residents from the tropical diseases emanating from the black town. The Committee emphasised the need for improved drainage, larger tanks, new aqueducts and bridges and cleaner waterfronts. The sanitary engineering projects in particular areas of the city reflected the concern regarding the close link between cleanliness and health.^62^ An Improvement Committee surveyed the city and suggested measures to improve drainage, water courses and streets. The Lottery Committee was eventually replaced by the Fever Hospital Commission in 1836 that identified and recommended restructuring the hotbeds of epidemic diseases within the city especially by improving drainage.^63^ The reports of sanitation surveys from this period framed Calcutta within a paradigm of backwardness, consolidating its image as a ‘pathological space’. Finally, powerful visual representations of the city in colonial documents helped create tropes of disease-ridden Indian localities and a symbolic geography of filth.^64^ Thus, the European disgust for filth created a context for the colonial state to regulate spaces and communities.

In 1845, Dr John Macpherson of the Bengal Medical Service enumerated several causes for seamen’s death in the city: ‘reckless’ behaviour, condition of stomach after long voyages, consumption of spirituous and poisonous liquor, compulsion of working under the midday sun, maundering in the markets during daytime, debauchery, sleeping out in the dew or on the damp deck, and living close to the mouths of sewers where their vessels were moored.^65^ Although not the leading cause of death among British seamen, cholera had a remarkably high mortality rate. In the previous year, only seventeen of the 371 seamen admitted in the Howrah General Hospital were affected by cholera. When it came to mortality, however, as high as ten of the seventeen cholera patients died, most of them within 9 hours of admission. In comparison, only nine other seamen died in the entire year, from common maladies such as dysentery, remittent fever, the ‘infrequent’ acute rheumatism, the ‘mild’ syphilis and the ‘very rare’ scurvy. The hospital records say that most cholera-related admissions (thirteen) and deaths (eight) were in April, which probably originated in the same ship.^66^ For 2 years, the hospital tried known remedies such as hot bath, which appeared to help in one case when combined with a number of other methods. But the problem of overcrowding and seamen’s tendency to turn up in the hospital at the last possible moment undermined the effectiveness of treatment.

Macpherson drew attention to British seamen’s greater vulnerability to cholera than other members of the colonial ruling race when he said that the seamen’s hospital and Howrah General Hospital had more cholera patients than the military hospital. As most merchant vessels were without surgeons, ailing seamen often had to wait for the captain to have some time off work to check on them. They lay helpless on the damp deck for the night, too weak to shout for help, waiting for other crew members to notice and send them to the nearest hospital on shore. Some of them died before or were near death by the time they were taken to the hospital. Seven out of ten died within 9 hours of admission to the Howrah Hospital. The treatment in hospitals was more effective than on board ship, but hardly adequate on account of the shortage of staff and beds. In most situations, patients received no formal treatment. Some were given hot baths when on the verge of collapse. Macpherson noticed that the severest cases often came from the same ship. His usual method of treatment was fifteen grains of sugar of lead combined with small quantities of opium, other than hot bath.^67^ The equipment suggests his access to medicine was probably very limited, as surgeons in Britain or even ship captains with medicine chests seemed to have more varieties of medicine at their disposal. It shows how little the colonial state invested in medical infrastructure intended for poor whites. The records of the private hospitals maintained by merchant shipping companies such as the P&O rarely mention how doctors treated their patients.

Other British surgeons were known for modifying remedies prescribed in British medical textbooks, integrating elements from indigenous therapeutics and experimenting on patients whose symptoms did not match conventional knowledge. For treating cholera, they used black pepper, calomel, ginger and asafoetida with opium, brandy or arrack and later cholera pills made of equal portions of opium, black pepper and asafoetida.^68^ The use of Indian ingredients such as black pepper, ginger and arrack in combination with European-origin calomel and brandy shows an eclectic exchange of medical ideas between Britain and India. Medical treatises on seamen’s health say nothing of consulting Indian pharmacopoeia though the latter’s influence on British maritime medicine was evident. The therapeutic treatments were understandably not standardised, as cholera pathology was uncertain till the end of the nineteenth century. Surgeons relied on early detection and start of treatment for cholera patients.^69^ The piecemeal evidence on cholera treatment in hospitals in Calcutta and other Indian port cities did not allow a more comprehensive evaluation of medical innovations, the sanitary environment for treatment and the impact of climate on the convalescence of patients. The ad hoc nature of administration, underfunding and limited number of medical staff indicate seamen’s hospitals might not have provided ideal nursing care in a hygienic building. It is amply evident from Macpherson’s account that seamen’s vulnerability to cholera was well-known, and it is to the sanitary measures undertaken in the port city to protect seamen that the next section will turn.

## The sanitary reform of sailortown

The discourse of poor health and sanitation associated with the sailortown areas of Indian port cities necessitated sanitary regulation of seamen’s living conditions and drinking habits. Historian Ishita Pande has argued that the threat of cholera had made certain colonial cities an object of sanitary intervention. Surgeons and medical superintendents of hospitals in Calcutta examined the disease’s topographic understanding and generated detailed statistics on disease prevalence, hospital admission and mortality.^70^ Pande says that by emphasising the need to make the city more sanitary and healthier, sanitary reformers championed the liberal colonial project of civilising ‘backward’, ‘ignorant’ and ‘unclean’ natives. A sense of responsibility for the city’s native inhabitants, who often failed to understand the benefits of sanitation, guided the plan to bring the entire city and not only the European inhabited parts under sanitary regulation.^71^ Cholera influenced this decision to a large extent. The fear and panic surrounding the cholera pandemics encouraged the municipal administration to probe the disease’s pathology and take sanitary measures.

The Bengal government followed the British model of adequate drainage, water filtration and sewage disposal. In Britain, provision of clean and pure water became an essential part of sanitation and health reforms in the 1840s and 1850s as the theory of cholera’s waterborne origin gained in popularity.^72^ The ideas of contagion and contamination undergirding many of these initiatives were most visible in the regulations placed upon the urban poor living in overcrowded and squalid habitats.^73^ In the 1850s, the Government of India started grappling with the idea that contamination and environmental conditions aided the spread of epidemics. Calcutta’s image as a disease hotbed provided a strong rationale to protect the port and the shipping industry that connected the colonial state with its homeland and was an important hub of global trade. Cholera did not strike in a regular pattern. Sometimes seamen visiting the relatively safe Esplanade or living on ships anchored away from the riverbank were cholera-stricken to a greater extent than seamen or ships located closer to the designated danger zones. In 1858, twenty-two ‘Protestant’ seamen died of cholera in the Sardinian frigate *Amazona* that was moored in the Esplanade. The rest of the seamen were evacuated to prevent further loss of life.^74^ Similarly, when cholera broke out on board *Hastings* and spread to other vessels in the port of Bombay, the entire crew were evacuated and the ships thoroughly cleaned.^75^ Shifting potential victims away from the site of cholera was a useful practice when hospitals overflowed with cholera patients.

The Bengal government started devising discernible plans of combating cholera in the 1860s. In a lecture at the Bethune Society in 1862, Dr Norman Chevers, Principal of the Calcutta Medical College, explained the importance of sanitation for preventing disease. He called sanitation ‘a mere matter of common sense’ informed by an instinctive quality of ‘self-preservation’.^76^ Calcutta was built upon a swamp rife with ‘malarious venoms’ of ague, dysentery, asthma and cholera. In the absence of proper drainage, the organic matter rotting in the vast salt lagoon next to the city polluted the air. Like many of his contemporaries, Chevers felt that sanitation in Calcutta was incomparably poor and propitious for pestilence. Supply of clean water was one of the most challenging engineering problems of the time. The water in the river was ‘frightfully impure’, filled as it were with 15 000 corpses and carcasses a year and about 40t of excreta daily.^77^ Utterly disappointed and worried about the state of sanitation, Chevers urged the Calcutta University to start a professorship of public health as part of the liberal education programme to teach the unknowing people in the ‘unhealthiest city’ in the ‘most improvable country in the world’.^78^ In 1864, Chevers published the most important tract on the health of European seamen in Calcutta, outlining the problems of cholera among other diseases for seamen temporarily staying in the city.^79^

Since many medical practitioners associated cholera with fluctuation in temperature and cold wind, government officials often thought that the cyclone in Calcutta in 1864 had changed the weather and aggravated the disease in the city.^80^ They believed certain localities such as Flag Street were a nursery of cholera. Chevers wrote that the Medical College Hospital, situated near Flag Street in the centre of the city, admitted more than twice as many sailors as the General Hospital, and most of these cases were from the street.^81^ Filth was considered the ‘visible representation’ of cholera.^82^ The use of unfiltered water by the local people for cooking and drinking posed a great challenge to public health. John Strachey, president of the Sanitary Commission of Bengal, commented that the ‘filthiness of Calcutta’ could not be compared with even the ‘filthiest quarters of the filthiest towns’ in India or other countries.^83^ He called Calcutta a ‘scandal’ and a ‘disgrace’ to a civilised government, where human corpses lay on the ground and floated in the river. He stated that as many as 5 000 human corpses, including 1 500 from government hospitals, were thrown into river.^84^ Strachey was not alone in expressing such views about the horrifying health and sanitary conditions of Calcutta. Similar observations had already been made in previous reports by health officers. The Sanitary Commission acknowledged neglect of the living conditions and health of British seamen in Calcutta. It noted with regret that the municipal corporation and the government had done little to alleviate the high percentage of mortality among seamen in city. Since they had not maintained proper registers, the statistics on mortality are not available.^85^ The Commission recommended comfortable accommodation and honourable amusement for seamen in the way the Sailor’s Home had done.^86^

Contemporary newspapers were critical of the neglect of British seamen. An anonymous author figuratively wrote that ‘drunkenness, disease, and death broods over the portals of Lall Bazaar’.^87^ Despite being located close to the city’s European quarter, this place was known for odour, filth, ‘dust, dirt and diseases’.^88^ Most of the city’s pubs were located on Lal Bazar, where the drains were mostly open and full of black putrid slime possibly accumulated for years. Newspapers criticised the ‘disgusting’ appearance of marketplaces, the irregularity of sanitary supervision and the failure to enforce sale of hygienic food. Although the popular white opinion considered dining at dingy eateries in this locality as below the dignity of Europeans, interactions between foreigners and locals continued unrestricted for a long time. Fatal disease rarely deterred seamen to spend time, ‘sit and boose and eat the air’ in the dens of gambling and drinking. The conditions were suitable for rapid circulation of epidemics. A major reason behind cholera was adulteration of liquor with poor quality water.^89^ Major G.B. Malleson wrote that such situations demanded constant care and vigilance by both the police authorities and the municipality.^90^ Chevers recommended better drainage along Flag Street to prevent accumulation of putrid water.^91^ By 1875, surgeons no longer associated cholera with exposure to extreme weather and drinking habits. They were now more concerned with the purity of drinking water, accepting Snow’s theory of waterborne cholera.

British seamen’s deplorable condition caused anxiety among the politically and socially conscious British at home and away, who became concerned about the young British men who travelled across continents to make their country prosperous. Especially the newspaper reports and the missionaries brought to notice the unacceptable living conditions of sailors in the hot tropical countries, in this case India. Such campaigning lent credence to seamen’s image as helpless victims and propagators of disease as ship after ship suffered from contagious or infectious diseases. Surgeons contended that seamen were not adequately supported by health authorities, which endangered not only seamen but also passengers and other people they encountered.^92^ Harry Leach, the resident medical officer of the hospital ship *Dreadnought*, said that British seamen’s ‘unsatisfactory’ condition had attracted lot of attention and was often discussed in periodicals. He pointed out that the scarcity of ‘competent’ sailors, coupled with the rise in wages, threatened the commercial interests of the merchant classes. Calling the Merchant Shipping Act 1854 a ‘maze’ of confusing clauses and amendments, he explored how it was abused by the shipowner, master, mate, as well as seamen. The Act failed to deliver the provisions of accommodation and health. The ‘sea as a service’ was so ‘unpopular’ that only 1% of Britain’s population were in seamanship. Therefore, naval and merchant ships employed inferior quality and foreign seamen, endangering the cargos, the ship and the British Empire.^93^ Leach considered endemic dysentery in India and China to be preventable and said that many seamen in Calcutta, Hong Kong and other Asiatic ports could be saved with proper care. He also suggested that seamen should avoid drinking ‘filthy mixtures of rum, arrack, sangaree’ to protect themselves from fatal consequences.^94^ In the 1860s, the municipal and port authorities in Calcutta realised the importance of supplying pure water to seamen and undertook several water and sewage management plans.

## Cholera and water impurity

As more seamen died of cholera, the quality of drinking water came under scrutiny. Cholera was by now linked with consumption of unfiltered water.^95^ A report in the *Indian Medical Gazette* in 1866 pointed out that each of the twenty-two sewers in Calcutta carried its refuse liquid and waste matter into the river Hooghly. It criticised Macpherson for ignoring that fact that the water seamen consumed from the river was full of these waste materials.^96^ On the contrary, he suspected a connection between the sewers and the disease, though he gave importance to location in his suggestion that ships stationed closer to the shore and near the mouths of the sewers were more susceptible to cholera.^97^ He stated the best way to protect seamen was to stop them from going to the shore (Calcutta port) before their outward journey. This was done several times, replicating the measure taken during an epidemic in Malta and at the Black Sea during the Crimean War. He concluded, ‘on the whole it may be said that sailors get Cholera by visiting Calcutta and lose it by going to the sea’. He said that ship captains were contented with drinking river water despite knowing about its polluted nature and ‘never’ complained about any health problem arising from drinking impure water. Strangely, no one suffering from chronic diarrhoea ever blamed the river water.^98^ Thus, we find that the *Indian Medical Gazette* wrongly pointed out that Macpherson attached little importance to using the water from the Hooghly as a cause of cholera.^99^ Rather, we find that Macpherson criticised seamen and ship captains for not realising the threat from the impurities contained in drinking water in Bengal that manifested during the journey back.^100^ The condition of drinking water seemed to have improved manifold as ship surgeons who were critical of almost every aspect of Calcutta praised the quality of its drinking water. The surgeon of HMS *Glasgow* wrote in 1875:

Whether the cholera poison be the product of animal or vegetable decomposition, or whether it be conveyed into the system through the alimentary or respiratory surfaces, Calcutta contains all the factors essential to its development and propagation, in abundance… 1st. Alluvial soil… always prolific in malarial emanations. 2nd. Insanitary condition of the native quarter… where the gutters are constantly overflowing with sewage matters… 3rd. Direct contamination of the river atmosphere, and indirectly that of the city, by gases of animal decomposition, emanating from the bodies of Hindoos thrown into it, or the parent river Ganges, for burial… 4th. The water supply, which we found very good, and free from organic impurities… The more ignorant of the natives cannot be made to comprehend the benefit conferred by pure water, and very frequently prefer drawing their supply from the nearest tank, the receptacle, in all probability, of all sorts of filth, sewage, and even cholera dejecta. Water thus procured is often used in the manufacture of soda water and other beverages sold in the bazaar, to men on leave, and there can be no doubt that most of the cases of cholera contracted by men-of-war’s men on shore, originate in this manner…the bazaar people are forbidden under heavy penalties to sell spirits or any other drink to soldiers. It would be a great boon if the same prohibition were applied to seamen.^101^

The report suggested all Indians and Europeans emulate the example of Bombay in the matter of drainage and supply of pure water, which the surgeon considered ‘the most potent antidotes of cholera’.^102^ In 1876, official reports downplayed the role of polluted water in precipitating cholera, arguing that the water consumed by the people of London was far more impure than the water of Calcutta. A chart in the Accounts and Papers of the House of Commons showed that the water in Calcutta contained 15.79 parts impurity in 100 000, whereas the figures for the water supplied by the Southwark Company and the New River Company in London in 1870 were 26.19 and 26.43, respectively. The report placed great emphasis on the problem of drainage in slum areas, where ‘all the refuse, both of the house and cook room, is thrown into open ditches filled with putrefying matter and a black greasy slime often several feet in depth’.^103^ The water sources in these areas were usually located near latrines, and thus easily contaminated. It was the wrong use rather than quality of water that caused health problems.

In a Memorandum in Vol. IX of the *India Office Sanitary Reports, 1876*, the Commission reported the total number of European, American, and Eurasian seamen in the port of Calcutta as 25 448 and the number of Asiatic seamen as 22 179. The report included a table of the main diseases and number of hospital admissions of the undistinguished category of ‘European seamen’. They were cholera (110), enteric fever (nine), malarious fever (244), simple fever (thirty-two), variola (none), sunstroke (three), scurvy (twenty-nine), dysentery (137), diarrhoea (148), liver disease (twenty-seven), venereal disease (133) and all other diseases (966), totalling 1 838 patients. The report specifically noted that fifty-three of the cholera patients died. It further mentioned that forty-six of the patients were from vessels anchored to the north of Fort Point, whereas fifty-five were from those anchored down south. The vessels on the lower part of the river were situated closer to where the sewage from Tolly’s Nullah and the Fort poured into the river, contaminating the water that was used for washing. The report put the blame on the suburban municipality for not adequately responding to the problem despite the Lieutenant Governor’s insistence. In 1876, the municipal water was used by 18 980 men on board 949 ships, nearly four times the figure in the preceding year.^104^ There were instances of river sewage seeping into the ship’s bilge and causing the outbreak of disease after the ship started its journey.

In the 1880s, when cholera was rife in Calcutta, it was often said in jest that drinking the ‘local firewater’ immunised seamen from the disease.^105^ A report in *Lancet* from 1887 that blamed the high death rate from cholera, around 11.1 per 1 000, among European sailors on ‘breathing its foul air, and partaking of drinks diluted not always with hydrant water’.^106^ It noted that such atrocious environment was rare. Yet, despite being cautioned, seamen continued using water from the river and other suspicious sources and falling victim to cholera. For instance, the second officer of the *Crofton Hall*, anchored at Budge Budge, prepared some fresh brine with water from the river to preserve beef on 20 January 1892. The crew began eating this beef as the ship left the port in the first week of June, and twenty-three out of the twenty-nine seamen immediately showed symptoms of cholera, six of them dying within a week. The ship turned back to Calcutta for want of enough seamen to complete the journey. Ten of the remaining affected seamen were sent to the General Hospital for treatment. The ailment was declared to be different from ‘true cholera’, but an examination of the brine in which the meat was preserved showed the presence of ‘atypical varieties’ of the cholera-inducing organism.^107^ Thus, cholera and its various manifestations imposed an overwhelming challenge of death and distress that could not be overcome before the beginning of the twentieth century.

## Conclusion

This article has charted the history of how the British naval authorities, ship surgeons and the port and municipal authorities tried to prevent cholera among British seamen in transit to Calcutta and in the Calcutta sailortown, uniquely associated with cholera in nineteenth century discourses on the disease. Through a study of seamen as sanitary subjects and disease carriers, it has generated several insights into maritime medicine and sanitary regulation in the nineteenth century. It has shed light on maritime medicine as a building block of the British naval authorities’ cholera prevention policy in the northern Indian Ocean region, expanding the scope of existing literature that had an exclusive focus on quarantine and movement of passengers and indentured labourers. By identifying a range of experiments with various forms of remedies and sanitary regulations, the article finds that surgeons on ships and on shore engaged with a variety of cholera pathologies, stressing miasma, putrefaction, contagion and transmissibility of pathogen through air or water. The use of unconventional treatments, in the absence of approved materials or when the surgeons could not match symptoms with textbook knowledge, indicates that situational demands countermanded medical dogma. Ship surgeons showed greater flexibility in their practice, not adhering steadfastly to either nervous or germ theories of disease, than many of their land-based colleagues. This discussion of cholera prevention and treatment on board ships deepens our understanding of the trajectory maritime medicine in the tropics and its importance relative to land-based medicine.

The article has expanded our knowledge of the regulation of ships, places and materials such as water. In the 1850s, the Government of Bengal adopted a sanitarian position in public health reform, giving importance to problems of dirt, disease, filth and sewage, raising the standards of cleanliness and hygiene and instituting sanitary engineering in port cities and sailortowns. The reforms had two aims: preventing seamen from being infected and infecting others and embedding Victorian standards of civilised living and public health. The impact of these reforms is, however, open to scrutiny. Most accounts indicate the river estuary rather than the city to have been the breeding ground of cholera, which is why the location of anchorage became an important issue for ships. Newspaper reports on the victimhood of British seamen reinforced the attention on Calcutta as a distinctively dangerous place. The waterborne theory of cholera influenced the municipal authorities to improve water filtration. Sanitary reforms focused on the squalid condition of the sailortown, poor drainage that led to decaying organic material mixing with water, and the paucity of clean water that compelled seamen to consume alcoholic beverages. The popularity of drinks that was known to cause cholera, diarrhoea and dysentery prompted the colonial state to regulate unlicensed liquor producers and vendors. Cholera epidemics were thus identified as the culmination of several racial, political and environmental factors.

## Footnotes

1 ‘Cholera in the Port’, *Indian Medical Gazette*, 1, 7 (1866), 190–1.

2

Tim Carter , Merchant Seamen’s Health, 1860–1960: Medicine, Technology, Shipowners and the State in Britain (Woodbridge: The Boydell Press, 2014).

3

William Macmichael , Is the Cholera Spasmodica of India a Contagious Disease? (London: John Murray, 1831).

4

Valeska Huber , ‘The Unification of the Globe by Disease? The International Sanitary Conferences on Cholera, 1851–1894’, The Historical Journal, 49, 2 (2006), 453–76.

5

Katherine Foxhall , Health, Medicine, and the Sea: Australian Voyages, c.1815–1860 (Manchester: Manchester University Press, 2012).

6 Carter, *op. cit.* (note 2), 80.

7

Jörg Vögele and Hideharu Umehara (eds), Gateways of Disease: Public Health in European and Asian Port Cities at the Birth of the Modern World in the Late 19th and Early 20th Century (Göttingen: Cuvillier Verlag, 2015).

8

A.B. Fry , ‘Cholera in Bengal, Past and Present’, Indian Medical Gazette, 60, 7 (1925), 301–7.PMC518872329010214

9

Norman Chevers , On the Preservation of the Health of Seamen (Calcutta: Military Orphan Press, 1864).

10

Mark Harrison , ‘A Question of Locality, The Identity of Cholera in British India, 1860–1890’, in David Arnold (ed.), Warm Climates and Western Medicine (Amsterdam: Rodopi, 1996), 133–598783624; Christopher Hamlin , ‘The Cholera Stigma and the Challenge of Interdisciplinary Epistemology, From Bengal to Haiti’, Science as Culture, 21, 4 (2012), 445–74.

11

Joseph Smith , The Claims of the British Seaman to the Protection of Parliament (London: C.A Bocking, 1850), 13.

12

Margaret Pelling , Cholera, Fever and English Medicine, 1825–1865 (Oxford: Oxford University Press, 1978).

13 For the history of cholera in the British Empire and India in the nineteenth century, see David Arnold , ‘Cholera and Colonialism in British India’, Past & Present, 113 (1986), 118–5110.1093/past/113.1.11811617906; Ira Klein, ‘Imperialism, Ecology and Disease, Cholera in India, 1850–1950’, *The Indian Economic & Social History Review,* 31, 4 (1994), 491–518; Jeremy D. Isaacs, ‘DD Cunningham and the Aetiology of Cholera in British India, 1869–1897’, *Medical History*, 42, 3 (1998), 279–305; Sheldon Watts, ‘From Rapid Change to Stasis, Official Responses to Cholera in British-ruled India and Egypt, 1860 to c. 1921’, *Journal of World History*, 12, 2 (2001), 321–74; Dhrub Kumar Singh, “Clouds of cholera’ and ‘clouds around cholera,’ 1817–70’, in Deepak Kumar (ed.), *Disease and Medicine in India: A Historical Overview* (New Delhi: Tulika, 2001), 144–65; Sheldon Watts, ‘Cholera and the Maritime Environment of Great Britain, India and the Suez Canal, 1866–1883’, *International Journal of Environmental Studies*, 63, 1 (2006), 19–38; Christopher Hamlin, *Cholera: The Biography* (Oxford: Oxford University Press, 2009); Pratik Chakrabarti, ‘Curing Cholera, Pathogens, Places and Poverty in South Asia’, *International Journal of South Asian Studies*, 3 (2010), 153–68; Projit Bihari Mukharji, ‘The ‘Cholera Cloud’ in the Nineteenth-Century ‘British World’: History of an Object-Without-an-Essence’, *Bulletin of the History of Medicine*, 86, 3 (2012), 303–32; Mark Harrison, ‘The Great Shift, Cholera Theory and Sanitary Policy in British India, 1867–1879’, in Biswamoy Pati and Mark Harrison (eds.), *The Social History of Health and Medicine in South Asia* (London: Routledge, 2018), 37–60; Mark Harrison, ‘A Dreadful Scourge, Cholera in Early Nineteenth-Century India’, *Modern Asian Studies*, 54, 2 (2020), 502–53.

14 South Asian pilgrims were ‘notorious for their professional filthiness’ and therefore viewed as ‘exceptionally dangerous vectors of transmission.’ Saurabh Mishra , Pilgrimage, Politics, and Pestilence: The Haj from the Indian Subcontinent, 1860–1920 (New Delhi: Oxford University Press, 2011), 54 . See also Valeska Huber , Channelling Mobilities: Migration and Globalisation in the Suez Canal Region and Beyond, 1869–1914 (Cambridge: Cambridge University Press, 2013).

15

David Arnold , ‘The Indian Ocean as a Disease Zone, 1500–1950’, South Asia, 14, 2 (1991), 10–11.

16 Sara Caputo, ‘Alien Seamen in the British Navy, British Law, and the British State, c. 1793–c.1815’, *The Historical Journal*, 62, 3 (2019), 685–707.

17 An exception was maritime surgeon William Hunter’s work on the diseases, health, mortality, and treatment including diet and exercise of lascars. The book contained several letters from the ship captains recounting the medical experiments and trials conducted on ships. William Hunter , An Essay on the Diseases Incident to Indian Seamen, Or Lascars, on Long Voyages (Calcutta: Honorable Company’s Press, 1804).

18

James Kennedy , The History of Contagious Cholera (London: James Cochrane, 1831), 239–40.

19
*Ibid.*, 199–200.

20

Mark Harrison , Contagion: How Commerce Has Spread Disease (New Haven: Yale University Press, 2009), 67.

21
*Ibid.*, 105.

22

Erwin H. Ackerknecht , ‘Anticontagionism between 1821 and 1867’, International Journal of Epidemiology, 38 (2009), 7–21.1918820210.1093/ije/dyn254

23

James Morss Churchill , ‘Contagiousness of Cholera’, The Lancet, 62, 1573 (1853), 393–94.

24 Sailing ships were not out of operation yet. In a lecture in November 1873, Lord Brassey, founder of the *Naval Annual* magazine, explored the transition from sail to steam and the great impact of steam vessels on British trade and commerce. Along with this technical and engineering advancement, the Suez Canal had considerably increased Britain’s maritime connection with the East. Steam shortened voyages, but sailing vessels were still used to collect coal and other raw materials from British acquired territories such as Gibraltar, Malta, Port Said and Aden and store them till a good business opportunity arose, thus serving as ‘conveyance’ and ‘warehouse’. Lord Brassey, *Mercantile Marine and Navigation from 1871 to 1894* (London: Longmans, Green & Co., 1894), 4–5.

25 Harrison, *op. cit.* (note 20), 140–41.

26

John Slight , The British Empire and the Hajj, 1865–1956 (Cambridge: Harvard University Press, 2015).

27 Harrison, *op. cit.* (note 20), 140–41.

28

Myron Echenberg , Africa in the Time of Cholera: A History of Pandemics from 1817 to the Present (Cambridge: Cambridge University Press, 2011).

29

Alison Bashford , Imperial Hygiene: A Critical History of Colonialism, Nationalism and Public Health (Basingstoke: Palgrave Macmillan, 2004), 142.

30

Heber Smith , ‘Sailors as Propagators of Disease’, in Public Health Papers and Reports 1873 (New York: Hurd and Houghton, 1875), 447.PMC227268919599878

31
*Ibid.*, 448.

32 Harrison, *op. cit.* (note 20), 30–31.

33

Michael Quinlan , ‘Precarious and Hazardous Work, The Health and Safety of Merchant Seamen 1815–1935’, Social History, 38, 3 (2013), 295.

34

Norman Howard-Jones , ‘Cholera Therapy in the Nineteenth Century’, Journal of the History of Medicine and Allied Sciences, 27, 4 (1972), 373–95.456335110.1093/jhmas/xxvii.4.373

35

Donald D. Vogt , ‘Trends in 19th Century American Cholera Therapy’, Pharmacy in History, 16, 2 (1974), 43–53.11609235

36
*The Seamen’s New Medical Guide* (Liverpool: Mitchell, Heaton and Mitchell, 1842), 113.

37
*Ibid.*, 113–4.

38

Edward Palmer , A Guide-Book to the Government Medicine Chest for Merchant Seamen (London: H. Silverlock, 1845), 42.

39
*Ibid.*, 115–16.

40

Peter Vinten-Johansen et al. Cholera, Chloroform, and the Science of Medicine: A Life of John Snow (Oxford: Oxford University Press, 2003), 250.

41

Amanda J. Thomas , The Lambeth Cholera Outbreak of 1848–1849: The Setting, Causes, Course and Aftermath of an Epidemic in London (Jefferson: McFarland, 2010), 48–9.

42

Gustavus R.B. Horner , Diseases and Injuries of Seamen (Philadelphia: Lippincott, Grambo & Co, 1854), 147.

43
*Ibid.*, 148.

44

Frederick D. Fletcher , The Seaman’s Medical Friend, 2nd edn (Liverpool: W. Fearnall, 1857), 58–9.

45

Harry Leach , The Ship Captain’s Medical Guide, 2nd edn (London: Simpkin, Marshall & Co., 1868), 43–4.

46 Curtis, aware of the constraints of care on board ship, said limited medication and lack of assistants hindered his experiments on patients. Charles Curtis , An Account of the Diseases of India: As They Appeared in the English Fleet, and in the Naval Hospital at Madras, in 1782–83 (Edinburgh: W. Laing, 1807), 58.

47 ‘Naval Intelligence’, *The Times*, 29 November 1852, 7.

48 Charles Forbes, *Journal of Further Voyages for the P. & O. Company between Bombay, Singapore and Hong Kong, and on a Return Voyage to England, August 1854–June 1855*, Wellcome Library, MS5959.

49
*Statistical Report of the Health of the Navy for the Year 1875*, 230–1.

50

Robert Lawson , ‘Observations on Outbreaks of Cholera in Ships at Sea’, Transactions of the Epidemiological Society of London, 3, 2 (1874), 297–9.PMC552358729418941

51
*Statistical Report of the Health of the Navy for the Year 1875* (House of Commons, 1876), 226.

52
*Ibid.*, 301.

53 The surgeon was referred to as Dr Tuloch in ‘Military and Naval Intelligence’, *The Times*, 29 September 1859, 10.

54
*Statistical Report of the Health of the Navy for the Year 1875*, 303–4.

55
*Ibid.*, 305.

56

Robert Lawson , ‘On the Manifestations of Cholera in Ships at Sea, and Their Connections with Outbreaks on Land’, Transactions of the Epidemiological Society of London 11 (1892), 84.PMC553395529419089

57

Theodore J. Preston , ‘Epidemic Disease in the Royal Navy’, Transactions of the Epidemiological Society of London, 14 (1895), 47.PMC554245729419180

58
*Ibid.*, 48.

59

Gavin Milroy , ‘Epidemiological Conclusions and Suggestions’, The British and Foreign Medico- Chirurgical Review, 46, 92 (1870), 472.PMC516320330162980

60

W.G. Romeril , ‘Sanitation in the Mercantile Marine’, Journal of the Sanitary Institute, 17, 2 (1896), 152–73.

61

Robert Home , Of Planting and Planning: The Making of British Colonial Cities, 2nd edn (London: Routledge, 2013), 68.

62

Partho Datta , Planning the City: Urbanization and Reform in Calcutta, c. 1800–c. 1940 (New Delhi: Tulika Books, 2012), 28–30.

63

Mark Harrison , Public Health in British India: Anglo-Indian Preventive Medicine (Cambridge: Cambridge University Press, 1994), 203.

64

Nabaparna Ghosh , A Hygienic City-Nation: Space, Community, and Everyday Life in Colonial Calcutta (Cambridge: Cambridge University Press, 2020), 8.

65

John Macpherson , ‘A Few Remarks on the Diseases of Seamen in the Year 1844’, Calcutta Journal of Natural History, 6, 22 (1845), 252.

66 In one case, the cause of cholera was supposedly as innocuous as drinking two tablespoons of cream of tartar in water.

67 Macpherson, *op. cit.* (note 65), 252.

68 Arnold, ‘Cholera and Colonialism’, *op. cit.* (note 13), 136.

69

J. Campbell Brown , Report on the Calcutta Medical Institutions for the Year 1871 (Calcutta: Bengal Secretariat Press, 1872), 14.

70

Ishita Pande , Medicine, Race and Liberalism in British Bengal: Symptoms of Empire (London: Routledge, 2010), 103.

71
*Ibid.*, 97–8.

72

John Broich , ‘Engineering the Empire, British Water Supply Systems and Colonial Societies, 1850–1900’, Journal of British Studies, 46, 2 (2007), 346.

73

Roy Porter , Disease, Medicine and Society in England, 1550–1860, 2nd edn (Basingstoke: Macmillan, 1993), 55–7.

74

David B. Smith , Report on the Drainage and Conservancy of Calcutta (Calcutta: Bengal Secretariat Press, 1869), 13.

75 ‘India’, *The Times*, 3 October 1849, 6.

76

Norman Chevers , The Sanitary Position and Obligations of the Inhabitants of Calcutta (Calcutta: R.C. Lepage, 1863), 1–2.

77
*Ibid.*, 19.

78
*Ibid.*, 27.

79 Chevers, *op. cit.* (note 9).

80

James Gastrell and Henry Blanford , Report on the Calcutta Cyclone of the 5th October 1864 (Calcutta: O.T. Cutter Military Orphan Press, 1866), 126.

81 Chevers, *op. cit.* (note 9), 39, 67.

82

Pratik Chakrabarti , ‘Purifying the River, Pollution and Purity of Water in Colonial Calcutta’, Studies in History, 31, 2 (2015), 186 . As Ernest Hart, Chairman of the National Health Society in Britain, remarked, ‘Cholera [was] a filth disease, carried by dirty people to dirty places, and there spread by dirt and the use of the dirty water. With pure water, pure air, pure soil, and pure habits, cholera need not be feared.’ Ernest Hart, ‘Cholera, where it Comes from and How it is Propagated’, *British Medical Journal,* 16, 96 (1893), 1.

83 John Strachey, ‘Minute by the President of the Sanitary Commission of Bengal of 1864’, quoted in David Boyes Smith, *Report on the Drainage and Conservancy of Calcutta* (Calcutta: Bengal Secretariat Press, 1869). For an understanding of the social and moral symbolism of ‘filth’ in Victorian England, see Lee Jackson , Dirty Old London: The Victorian Fight against Filth (New Haven: Yale University Press, 2015).

84
*First Report of the Sanitary Commission of Bengal 1864–65*, 61.

85
*Ibid.*, 66.

86 ‘Sailor Life in Calcutta’, *Calcutta Review,* 40 (1865), 466.

87
*Ibid.*, 465.

88 ‘Calcutta’, *Saturday Review of Politics, Literature, Science and Art,* 19, 496 (1865), 507.

89

Manikarnika Dutta , ‘European Sailors, Alcohol, and Cholera in Nineteenth-Century India,’ in Gwyn Campbell and Eva-Maria Knoll (eds.), Disease Dispersion and Impact in the Indian Ocean World (Cham: Palgrave Macmillan, 2020), 191–210.

90 G.B. Malleson, ‘The State of the Sailors in Calcutta’, British Library IOR, P/437/29, Government of India, Home Department Proceedings, Marine 1866, 18.

91 Chevers, *op. cit.* (note 9), 51.

92 Smith, *op. cit.* (note 30), 447–53.

93

Harry Leach , A Report on the Hygienic Condition of the Mercantile Marine, and on the Preventable Diseases of Merchant Seamen (London: Arthur M. Walker, 1867), 5–6.

94
*Ibid.*, 19–20.

95 Chevers, *op. cit.* (note 9), 41.

96 ‘Cholera in the Port’, *Indian Medical Gazette,* 1, 8 (1866), 225.

97
*Ibid.*

98

John Macpherson , Cholera in its Home, (London: John Churchill and Sons, 1866), 26–7.

99 ‘Cholera in the Port’, *op. cit.* (note 96), 226.

100 Macpherson, *op. cit.* (note 98), 28.

101
*Statistical Report of the Health of the Navy for the Year 1875*, 227–8.

102
*Ibid.*, 229.

103
*Reports on Sanitary Measures in India in 1876–77, Together with Miscellaneous Information up to June 1878, Vol. X* (London: Eyre and Spottiswoode, 1878), 184.

104
*Ibid.*, 185.

105

Stan Hugill , Sailortown (London: Routledge and Kevin Paul, 1967), 315.

106 ‘Health of Calcutta’, *The Lancet,* 130, 3349 (1887), 931–2.

107 ‘The Bacteriology of the “Crofton Hall” Tragedy’, *Indian Medical Gazette,* 27, 12 (1892), 374–5.

